# Enlarged periventricular space and periventricular lesion extension on baseline brain MRI predicts poor neurological outcomes in cryptococcus meningoencephalitis

**DOI:** 10.1038/s41598-021-85998-6

**Published:** 2021-03-19

**Authors:** Woo-Jin Lee, Young Jin Ryu, Jangsup Moon, Soon-Tae Lee, Keun-Hwa Jung, Kyung-Il Park, Manho Kim, Sang Kun Lee, Kon Chu

**Affiliations:** 1 Department of Neurology, Seoul National University Hospital, 101, Daehak-ro, Jongno-gu, Seoul, 110-744 South Korea; 2 Laboratory for Neurotherapeutics, Center for Medical Innovations, Biomedical Research Institute, Seoul National University Hospital, Seoul, South Korea; 3 Department of Radiology, Seoul National University Bundang Hospital, Seongnam, South Korea; 4 Rare Disease Center, Seoul National University Hospital, Seoul, South Korea; 5 Department of Neurology, Seoul National University Hospital Healthcare System Gangnam Center, Seoul, South Korea; 6 Protein Metabolism Research Center, Seoul National University College of Medicine, Seoul, South Korea

**Keywords:** Biomarkers, Neurology, Pathogenesis, Risk factors

## Abstract

In *Cryptococcus neoformans* meningoencephalitis, brain MRI findings might reflect the phathomechanism of disease progression that is fungal accumulation in the peri-venular space and consequent invasion into the parenchyma. This study analyzed serial brain MRI findings of 76 patients with cryptococcus meningoencephalitis in association with the disease progression and outcomes. MRI parameters included the enlarged periventricular space (ePVS) score (range 0–8), periventricular lesion extension, cryptococcoma, and hydrocephalus. Clinical outcomes at 2-week, 10-week, and 6-month were evaluated using modified Rankin scale (mRS). At 6 months, 15 (19.7%) patients died and 34 (44.1%) had poor neurological outcomes (mRS scores > 2). At baseline, an ePVS score of ≥ 5 (Odds-ratio [OR]: 94.173, 95% confidence-interval [95%CI]: 7.507–1181.295, *P* < .001), periventricular lesion extension (OR: 51.965, 95%CI: 2.592–1041.673, *P* = .010), and presence of encephalitis feature (OR: 44.487, 95%CI: 1.689–1172.082, *P* = .023) were associated with 6-month poor outcomes. Presence of two or more risk factors among encephalitis feature, ePVS score ≥ 5, and periventricular lesion extension at baseline, was associated with 6-month poor outcomes (area under the curve [AUC]: 0.978, *P* < .001) and mortality (AUC: 0.836, *P* < .001). Disease progression was associated with interval development of cryptococcoma and hydrocephalus. Brain MRI findings might be useful in predicting outcomes and monitoring the progression of cryptococcus meningoencephalitis.

## Introduction

*Cryptococcus neoformans* meningoencephalitis is a serious central nervous system (CNS) complication in immunocompromised patients and is associated with a high mortality rate^1–4^. Although protocols including the administration of intravenous amphotericin B combined with flucytosine for acute induction treatment and fluconazole for consolidation and long-term maintenance have been established as standard regimen^2–5^, the clinical outcomes are considerably heterogeneous and a significant portion of patients with mild baseline severity neurologically deteriorate and end up with death or permanent sequelae^1,3,6,7^. Prognostic factors for poor outcomes include old age, higher antigen titer in the cerebrospinal fluid (CSF), larger ex vivo capsule size of the fungus, increased or decreased intracranial pressure (ICP), high peripheral white blood cell (WBC) count, low body weight, anemia, and features that constitute encephalitis such as reduced Glasgow coma scale (GCS) scores or presence of a seizure. However, as the main outcome parameters in those previous studies were mortality, antigen clearance from the cerebrospinal fluid, or crudely the cure of the disease, which do not account for the neurological outcomes^1,8,9^. One study evaluated cognitive outcomes for the patients who survived from cryptococcus meningitis, but it did not measure the overall neurological outcome^7^. Therefore, a marker that reflects the disease pathomechanism and estimates the risk of disease progression and poor neurological outcome is still lacking^6,7^.

The major route of entry of Cryptococcus into the CNS might be the key to explain the mechanism of disease progression and subsequent poor outcomes. Leukocyte-bound or free Cryptococci can exit the small-sized vessels in the brain and are accumulated in the perivascular space of the CNS, especially the peri-venular space^10^. Considering that the peri-venular space lacks pial membrane^11^, it can be postulated that the degree of peri-venular flow stagnation caused by the accumulated Cryptococcus might determine the risk of Cryptococcus invasion into the brain parenchyma, manifesting as the progression of disease^4,10^.

Enlarged perivascular space (ePVS) is a common brain magnetic resonance imaging (MRI) feature associated with Cryptococcus meningoencephalitis^12,13^. Given that ePVS might reflect the perivascular CSF flow stagnation caused by Cryptococcus accumulation, its degree might predict the risk of disease progression and poor outcomes. Similarly, other MRI findings such as parenchymal cryptococcoma or hydrocephalus might be utilized to monitor the neurological deterioration due to disease progression^12,13^.

In this study, we hypothesized that brain MRI findings might reflect the pathomechanism underlying disease progression and predict the outcomes of Cryptococcus meningoencephalitis, and analyzed the brain MRI findings, their serial changes, and its association with the disease progression and outcomes.

## Materials and methods

### Study subjects

This retrospective cohort study initially included all consecutive individuals admitted to the neurology department of the Seoul National University Hospital between January 2000 and December 2019 who were diagnosed with Cryptococcus meningoencephalitis. Among the initially included 117 individuals, the final study population was defined according to the following criteria: (1) underwent baseline brain MRI evaluation; (2) availability of clinical, treatment, laboratory, and long-term (> 6 months) neurological outcome data. According to the criteria, 33 patients without brain MRI evaluations and eight with inadequate data were sequentially excluded and the remaining 76 individuals were included in the study analysis. Diagnosis of Cryptococcus meningoencephalitis was based on the detection of the Cryptococcus antigen in CSF by latex agglutination or by lateral flow assay along with or without detecting Cryptococcus in CSF by culture or India ink assay^2,14,15^. The design of this study was approved by the institutional review board of the Seoul National University Hospital (SNUH) and the study was performed in compliance with the SNUH IRB regulations and the International Conference on Harmonisation guideline for Good Clinical Practice. Informed consent was waived by the IRB, as this study did not intervene with the evaluation or treatment process of the patients, and the patients were anonymized during the study process.

### Clinical and laboratory evaluation

Along with the demographic information, patients’ underlying immune status was reviewed and the causes of immunodeficiency were categorized as follows: Human Immunodeficiency Virus (HIV) infection, hematologic malignancy, solid organ cancer, post-transplant status, and long-term use of high-dose immune suppressants (for indications other than cancer treatment or post-transplantation immunosuppression)^4,16^. At baseline, encephalitis feature was defined according to the 2013 Consensus Statement of the International Encephalitis Consortium diagnostic criteria as: (1) altered mental status lasting more than 24 h without an alternative cause and (2) 3 or more of the followings: documented fever (> 38.0 °C); seizures not fully attributable to a preexisting seizure disorder; new onset of focal neurologic findings; CSF WBC count ≥ 5/mm^3^; and abnormal brain MRI findings suggestive of encephalitis^17^. Baseline GCS score and modified Rankin Scale (mRS) score data were also obtained from the patients’ medical records^1^. mRS is a widely used clinical scale for measuring the neurological function and the degree of disability in the daily activities after a stroke or other neurological diseases^18^. mRS ranges from 0 to 6, and each score represents neurological status as follows: 0, no symptoms or disabilities; 1, no significant disability despite some symptoms, which is able to perform all daily activities; 2, slight disability, which is able to carry out own daily activities without assistance, but not all previous activities; 3, moderate disability, which requires some help, but is able to walk unassisted; 4, moderately severe disability, which is unable to carry out own basic daily activities without assistance and unable to walk unassisted; 5, severe disability with bedridden status, which requires constant nursing care and attention; and 6, dead^18^. Nowadays, mRS is also widely used in measuring outcomes for encephalitis^19–23^.

CSF analysis included the evaluation of protein levels, WBC counts, and the elevation in the opening pressure (≥ 20 cmH2O)^8^. CSF Cryptococcus antigen titer was evaluated semi-quantitatively, and high antigen titer was defined as antigen detection in > 1:1 000 dilution^1^.

### Treatment profile analysis

Intravenous amphotericin (0.7–1.0 mg/kg/day) with or without flucytosine (100 mg/kg/day) or fluconazole (400–800 mg/kg/day) was used for the induction treatment period (within 2 weeks from the treatment initiation). Oral fluconazole was used during the consolidation (8 weeks after the induction treatment) and long-term maintenance treatment period in most patients (74/76, 97.4%)^2–5^. Treatment profiles with the durations of each treatment regimen were reviewed.

### Outcome analysis

The scores on mRS was obtained at the time of treatment initiation, at 2 weeks, 10 weeks, and at 6 months. As a primary outcome, a mRS score of > 2 was designated as 6-month poor neurological outcome, according to the previous studies investigated the neurological outcomes in encephalitis^19–23^. Serial follow-up CSF data at 2 weeks (window time of ± 3 days), at 10 weeks (window time of ± 1 week), and at 6 months (window time of ± 1 month) were obtained if available. Antigen clearance was defined as negative conversion of CSF Cryptococcus antigen evaluated at 2 weeks and at 10 weeks.

### Magnetic resonance imaging analysis

Baseline and follow-up MRI were performed using 1.5-T or 3.0-T units with the protocols including T1/T2-weighted images and fluid-attenuated inversion recovery (FLAIR) sequences. Baseline and follow-up images were obtained from the same scanner. T1/T2-weighted images and FLAIR were obtained with the parameters as follows: slice number = 24–30, slice thickness/gap = 4.0–5.0/0.0–2.0 mm, repetition time/echo time = 6000–10,002/92–168.5 ms, field-of-view = 285–220 × 285–220 mm, and matrix = 220–512 × 192–400 (Supplemental Table 1 for the detailed MRI machines and parameters).

All the image analysis was separately performed by a neurologist (W-J L., 9 years of experience) and a radiologist (Y R., 9 years of experience), blinded to all clinical data. Consensus was made by discussion for the discrepant cases. ePVS was defined as small, sharply delineated ovoid or linear lesions with T2- hyperintensity and T1- hypointensity. ePVS was semi-quantitatively rated in two brain regions—basal ganglia (BG) and centrum semiovale (CS)—using the scale introduced by Wardlaw et al., ranging 0–4 (0 for no, 1 for 1–10, 2 for 11–20, 3 for 21–40, and 4 for > 40 ePVS). BG- and the CS-ePVS scores were summed up to a total ePVS score ranging 0–8^24^.

**Figure 1:**
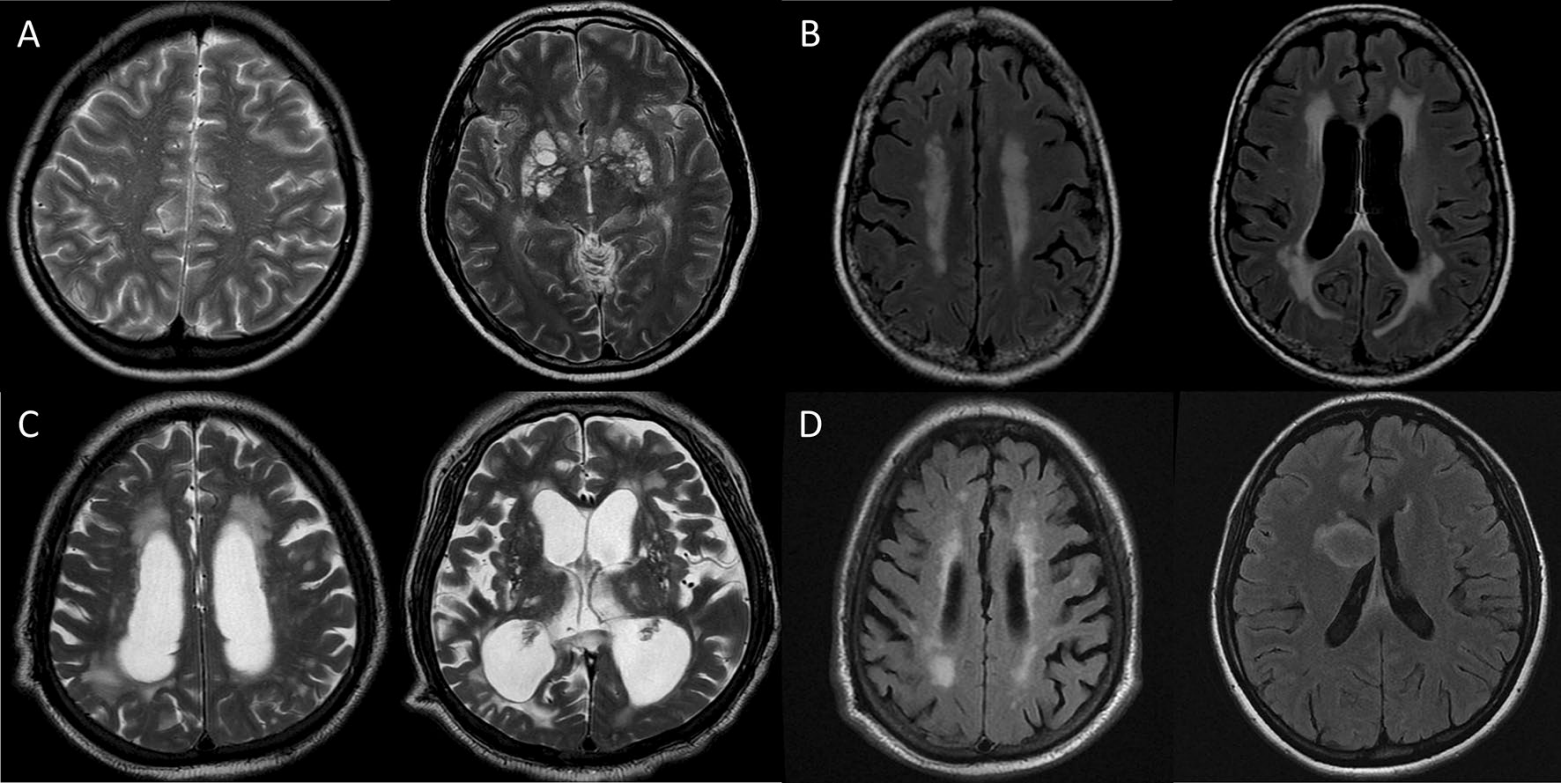
Examples of the brain MRI findings associated with cryptococcus meningoencephalitis. Panel (**A**), enlarged periventricular space (ePVS) in centrum semiovale (> 40 EPVS, score = 4, left panel) and basal ganglia (> 40 EPVS, score = 4, right panel); Panel (**B**), periventricular extension of the T2 high-signal intensity (HSI) on T2 fluid attenuated inversion recovery (FLAIR) images; Panel (**C**), hydrocephalus (Evans ratio = 0.42); and Panel (**D**), development of multiple cryptococcomas in right parietal cortex (left panel) and right caudate nucleus (right panel, T2 FLAIR images).

Periventricular lesion extension was defined as T2/FLAIR hyperintensity along the lateral ventricular border extending to the periventricular or subcortical parenchyma. Cryptococcoma was defined as single or multiple discrete T2/FLAIR hyperintensity lesions with T1 hypointensity in brain parenchyma^24^. Hydrocephalus was defined as the Evans’ index (the ratio of the maximal frontal horn width of lateral ventricle to the transverse inner skull diameter) of ≥ 0.3 (Fig. 1). For the 59 (77.6%) baseline MRIs with available T1 contrast enhancement images, the presence of an evident pathologic contrast enhancement in the brain parenchyma was also evaluated. To evaluate the serial changes in the MRI parameters, follow-up MRIs at 2 weeks (window time of ± 3 days), at 10 weeks (window time of ± 2 weeks), and at 6 months (window time of 5–10 months) were analyzed, if available.

### Statistical analysis

SPSS 25.0 (SPSS Inc., Chicago, IL, USA) was used for the statistical analyses. Data were reported as numbers (percentages), means ± standard deviations, or medians [interquartile ranges, IQR]. In univariate analyses, Pearson’s chi-square test and Student’s *t*-test were used. To evaluate factors associated with a poor neurological outcome, logistic regression analyses were performed including the parameters with a *P* < .10 in univariate analyses using a backward elimination method. Age was included in the final model of every regression analysis. Regression analyses were also performed separately for the subgroup without an encephalitis feature at baseline. Regression analysis for mortality was not performed due to its low frequency. Receiver operating characteristic (ROC) curve were drawn to evaluate the prognostic value of the factors derived from the regression analysis and to designate a cut-off value for predicting a poor outcome. For every analysis, a *P* value < .05 was considered statistically significant. Inter-reader reliability for the MRI parameters were evaluated using Cohen’s κ values.

## Results

Among the 76 patients (28 [36.8%] women, 48 [63.2%] men, mean age 55.4 ± 14.9 years [range, 19–80 years]), 51 (67.1%) patients were immune compromised and 25 (32.9%) were immune competent. At baseline, 22 (28.9%) patients presented with encephalitis. In baseline MRI, median BG-, CS-, and total ePVS scores were 2 [1–3], 2 [0–3], and 4 [1–6], respectively. Periventricular lesion extension was found in 41 (53.9%), cryptococcoma in 12 (15.8%), and hydrocephalus in 10 (13.2%) patients. Evident pathologic contrast enhancement in the brain parenchyma was found in 3 (5.1%) of the 59 patients with available T1 contrast enhancement images. Due to the significant portion of patients without baseline T1 contrast enhancement images and the low frequency of evident pathologic contrast enhancement in the brain parenchyma, this parameter was excluded from further outcome analysis. Inter-rater reliability was 0.97 (95% CI: 0.95–1.00) for a baseline ePVS score of ≥ 5, 0.95 (95% CI: 0.92–0.98) for a periventricular lesion extension, 0.96 (95% CI: 0.93–0.99) for a cryptococcoma, and 0.99 (95% CI: 0.95–1.00) for a hydrocephalus. The median time to treatment initiation was 3 [2–7] days from the onset of symptoms. For induction treatment, amphotericin was used in 76 (100.0%), flucytosine in 43 (56.6%), and fluconazole in 23 (30.3%) patients. For consolidation and maintenance, fluconazole was used in 74 (97.4%) patients. Amphotericin was maintained for 30 [20.3–42], flucytosine for 19 [10.5–31.5], and fluconazole for 110 [60–223] days.

The cumulative number of patients who achieved CSF antigen clearance at 10 weeks was 50 (65.8%). At 6 months, median mRS score was 2 [0–4] and mortality rate was 15 (19.7%). Detailed clinical, CSF, baseline MRI, treatment, and outcome profiles are described in Table 1. Numbers of patients with magnetic resonance image (MRI) evaluations included at each time points were 76, 40, 49, and 59 at baseline, 2-week, 10-week, and 6-months, respectively.

**Table 1:** Comparison of the clinical, laboratory, and treatment profiles between the groups with or without poor outcomes.

	Total (n = 76)	Good 6-month outcome (n = 42)	Poor 6-month outcome (n = 34)	*P*
**Demographic and clinical profiles**				
Female sex (%)	28 (36.8)	14 (33.3)	14 (41.2)	.387
Age of onset (years)	55.4 ± 14.9	52.9 ± 16.6	58.6 ± 12.2	.117
**Underlying immune status**				
Compromised	51 (67.1)	31 (73.8)	20 (58.8)	.324
HIV infection	19 (25.0)	11 (26.2)	8 (23.5)	.895
Hematologic malignancy	12 (15.8)	9 (21.4)	3 (8.8)	.165
Solid organ cancer	9 (11.8)	5 (11.9)	4 (11.8)	.948
Post-transplant	4 (5.3)	2 (4.8)	2 (5.9)	.788
High-dose immune suppressant	7 (9.2)	4 (9.5)	3 (8.8)	.975
Immune competent	25 (32.9)	11 (26.2)	14 (41.2)	.324
Fever > 38.0 °C	70 (92.1)	40 (95.2)	30 (88.2)	.264
GCS < 15	40 (52.6)	18 (42.9)	22 (64.7)	.095
Seizure	7 (9.2)	2 (4.8)	5 (14.7)	.147
Encephalitis	22 (28.9)	2 (4.8)	20 (58.8)	< .001**
mRS score	2 [1–3]	1 [1–2]	3 [2–4]	< .001**
**CSF profiles**				
CSF protein level (mg/dL)	75 [59–118]	69 [56–80.6]	84 [67.5–202.5]	.079
CSF WBC count (/uL)	46.5 [8.3–137.3]	68 [8–164.8]	38 [11–82.5]	.482
CSF opening pressure (≥ 20 cm H_2_O)	32 (42.1)	10 (23.8)	22 (64.7)	< .001**
High Ag titer (> 1000)	2 (2.6)	0 (0.0)	2 (5.9)	.160
**MRI profiles**				
BG ePVS score	2 [1–3]	1 [0–2]	3 [2–3]	< .001**
CS ePVS score	2 [0–3]	0 [0–2]	3 [3–4]	< .001**
Total ePVS score	4 [1–6]	1 [0–3.3]	6 [5–7]	< .001**
Periventricular lesion extension	41 (53.9)	10 (23.8)	31 (91.2)	< .001**
Cryptococcoma	12 (15.8)	1 (2.4)	11 (32.4)	.01**
Hydrocephalus	10 (13.2)	1 (2.4)	9 (26.5)	.22*
**Treatment profiles**				
Induction treatment				
Amphotericin	76 (100.0)	42 (100.0)	34 (100.0)	1.000
Flucytosine	43 (56.6)	23 (54.8)	20 (58.8)	.541
Fluconazole	23 (30.3)	14 (33.3)	9 (26.5)	.317
Fluconazole consolidation + maintenance treatment	74 (97.4)	40 (95.2)	34 (100.0)	.425
**Outcome profiles**				
Antigen clearance at 2-weeks	28 (36.8)	25 (59.5)	3 (8.8)	< .001**
Antigen clearance at 10-weeks	50 (65.8)	37 (88.1)	13 (38.2)	< .001**
mRS score at 10-weeks	2 [0–4]	0 [0–1]	5 [4–6]	< .001**
Mortality at 10-weeks	10 (13.2)	0 (0.0)	10 (29.4)	.004**
mRS score at 6-months	2 [0–4]	0 [0–0]	5 [4–6]	< .001**
Mortality at 6-months	15 (19.7)	0 (0.0)	15 (44.1)	< .001**

Data are reported as mean ± standard deviation, or as median [interquartile range, IQR].

*HIV* human immunodeficiency virus, *GCS* Glasgow coma scale, *mRS* modified Rankin scale, *CSF* cerebrospinal fluid, *WBC* white blood cell, *ePVS* enlarged perivascular space.

*P < .05 and **P < .01.

In the univariate analysis, poor 6-month outcome was associated with baseline encephalitis feature (*P* < .001), elevated CSF opening pressure (*P* < .001), the presence of periventricular extension (*P* = .001), cryptococcoma (*P* = .022), hydrocephalus (*P* < .001), and higher scores of GB-, CS-, and total ePVS (all *P* < .001) in the baseline MRI. Demographics, underlying immune status, treatment profiles were comparable between the groups with poor or good outcomes (Table 1). Subsequently, logistic regression analysis indicated that total ePVS score (Odds ratio [OR]: 5.068, 95% CI: 1.627–15.785 for 1 score increment, *P* = .005) was independently associated with a poor 6-month outcome. The association of the periventricular lesion extension was marginal (*P* = .056). When total ePVS score was dichotomized, an ePVS score of ≥ 5 (OR: 94.173, 95% CI: 7.507–1181.295, *P* < .001), baseline encephalitis feature (OR: 44.487, 95% CI: 1.689–1172.082, *P* = .023), and periventricular lesion extension (OR: 51.965, 95% CI: 2.592–1041.673, *P* = .010) were all independently associated with a poor 6-month outcome (Table 2).

**Table 2:** Logistic regression models for poor 6-month outcomes.

	Odd ratio (95% CI)	*P*	R^2^
**Model 1**			0.875
Age (years)	0.951 (0.854–1.059)	.358	
Encephalitis	47.271 (0.221–10,111.603)	.159	
ePVS score	5.068 (1.627–15.785)	.005**	
Periventricular extension	13.093 (0.924–185.574)	.057	
**Model 2**			0.877
Age (years)	0.968 (0.876–1.070)	.524	
Encephalitis	44.487 (1.689–1172.082)	.023*	
ePVS score ≥ 5	94.173 (7.507–1181.295)	< .001**	
Periventricular extension	51.965 (2.592–1041.673)	.010*	

*ePVS* enlarged perivascular space.

*P < .05 and **P < .01.

In the univariate analysis for the factors associated with 6-month mortality, the mortality of 6-month was associated with baseline encephalitis feature (*P* = .013), elevated CSF opening pressure (*P* = .032), periventricular lesion extension (*P* < .001), cryptococcoma (*P* = .013), and higher scores of GB-, CS-, and total ePVS (all *P* < .001) in the baseline MRI (Table 3). Due to low frequency (n = 15), multivariate analysis for mortality was not performed.

**Table 3:** Univariate analysis for the factors associated with 6-month mortality.

	6-month survivor (n = 42)	6-month non-survivor (n = 15)	*P*
**Demographic and clinical profiles**			
Female sex (%)	22 (36.1)	6 (40.0)	.781
Age of onset (years)	55.2 ± 15.9	56.4 ± 10.4	.717
**Underlying immune status**			
Compromised	41 (67.2)	11 (73.3)	.653
HIV infection	17 (27.9)	2 (13.3)	.189
Hematologic malignancy	11 (18)	1 (6.7)	.181
Solid organ cancer	3 (4.9)	4 (26.7)	.153
Post-transplant	5 (8.2)	1 (6.7)	.789
High-dose immune suppressant	6 (9.8)	1 (6.7)	.708
Immune competent	20 (32.8)	4 (26.7)	.653
Fever > 38.0 °C	57 (93.4)	13 (86.7)	.390
GCS < 15	30 (49.2)	10 (66.7)	.230
Seizure	5 (8.2)	2 (13.3)	.544
Encephalitis	13 (21.3)	9 (60)	.013*
mRS score	1 [1–3]	3 [2–4]	.005**
**CSF profiles**			
CSF protein level (mg/dL)	73.5 [59.3–97.9]	100 [50–196]	.810
CSF WBC count (/uL)	58 [12–144]	21 [0–135]	.402
CSF opening pressure (≥ 20 cm H_2_O)	22 (36.1)	10 (66.7)	.032*
High Ag titer (> 1000)	1 (1.6)	1 (6.7)	.475
**MRI profiles**			
BG ePVS score	2 [0–2]	3 [3–3]	< .001**
CS ePVS score	2 [0–3]	3 [3–4]	< .001**
Total ePVS score	3 [0–5]	6 [5–7]	< .001**
Periventricular lesion extension	27 (44.3)	14 (93.3)	< .001**
Cryptococcoma	5 (8.2)	7 (46.7)	.013*
Hydrocephalus	6 (9.8)	4 (26.7)	.193
**Treatment profiles**			.769
Induction treatment			
Amphotericin	42 (100.0)	15 (100.0)	1.000
Flucytosine	34 (55.7)	9 (60.0)	.769
Fluconazole	17 (27.9)	6 (40.0)	.558
Fluconazole consolidation + maintenance treatment	59 (96.7)	15 (100.0)	.484

Data are reported as mean ± standard deviation, or as median [interquartile range, IQR].

*HIV* human immunodeficiency virus, *GCS* Glasgow coma scale, *mRS* modified Rankin scale, *CSF* cerebrospinal fluid, *WBC* white blood cell, *ePVS* enlarged perivascular space.

*P < .05 and **P < .01.

We compared the clinical profiles and the outcomes between the groups with or without baseline encephalitis feature. The group with baseline encephalitis feature was associated with a higher frequency of HIV infection (*P* = .015), GCS score < 15 (*P* < .001), elevated CSF opening pressure (*P* < .001), the presence of periventricular extension (*P* < .001), cryptococcoma (*P* = .015), and hydrocephalus (*P* = .021) in the baseline MRI, and lower baseline mRS scores and higher scores of GB-, CS-, and total ePVS, compared to the group without encephalitis feature (all *P* < .001). The 6-month mRS score was also lower in the subgroup with baseline encephalitis feature (*P* < .001, Supplemental Table 2).

In the regression analysis to evaluate the factors associated with poor 6-month outcomes in the subpopulation without baseline encephalitis feature, total ePVS score (OR: 4.331, 95% CI: 1.457–12.875 for 1 score increment, *P* = .008) was associated with a poor 6-month outcome. In the model with dichotomized ePVS value, ePVS score ≥ 5 (OR: 60.073, 95% CI: 5.152–700.485, *P* = .001) and periventricular lesion extension (OR: 23.106, 95% CI: 2.796–297.176, *P* = .016) were significantly associated with a poor 6-month outcome (Supplemental Table 3).

**Figure 2:**
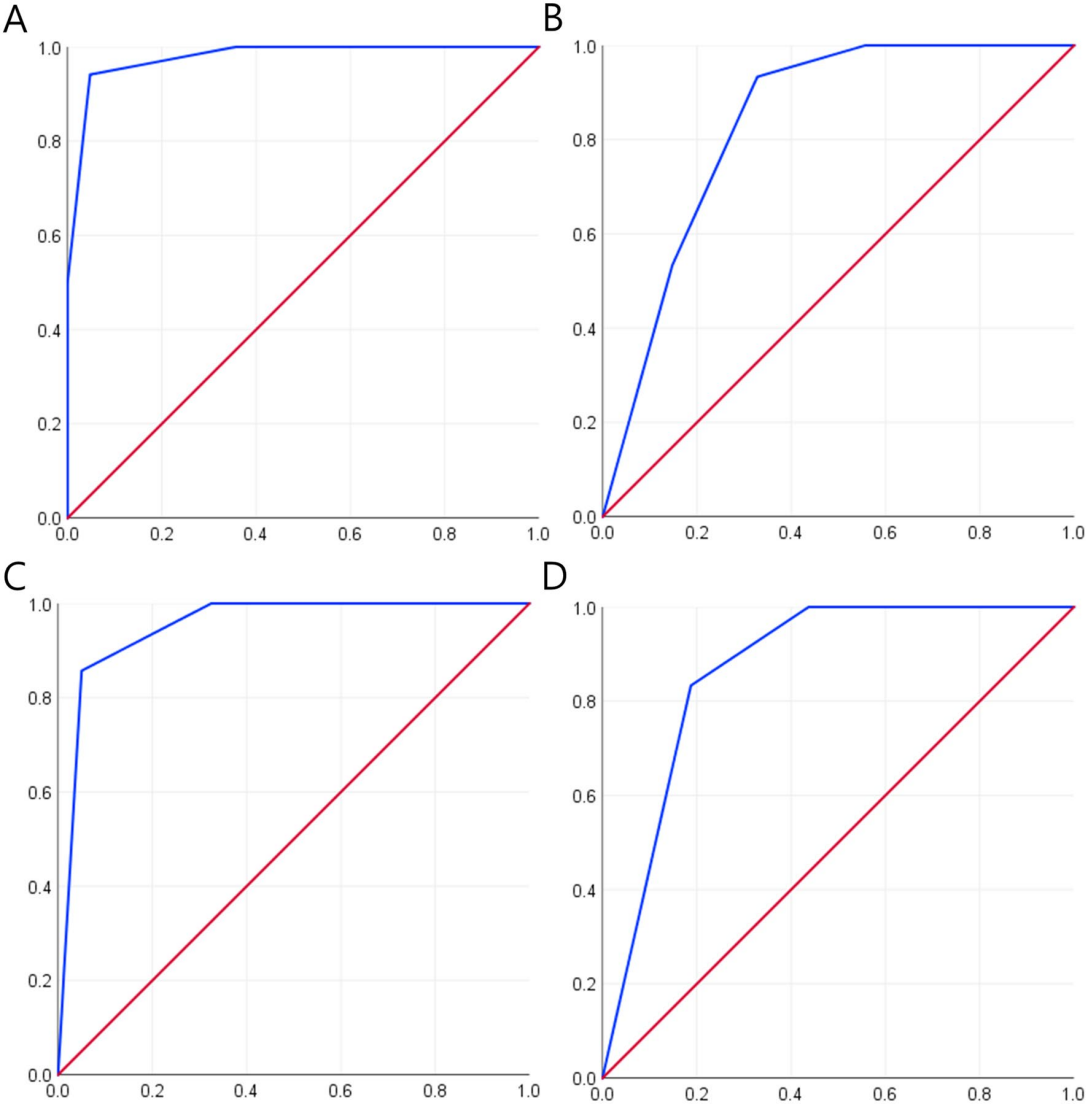
Receiver operating characteristic (ROC) curve analysis for a poor neurological outcome and mortality at 6-month. Receiver operating characteristic (ROC) curves for the association of the risk score with a poor neurological outcome (modified Rankin score > 2) and with mortality at 6-month in the total study population (Panels (**A**) and (**B**), respectively) and in the subgroup without baseline encephalitis feature (Panels (**C**) and (**D**), respectively). In each panel, blue lines indicate the ROC curve and red lines indicate the reference line.

Risk score for a poor 6-month outcome was calculated by summing up the number of the factors associated with poor outcomes (encephalitis feature, ePVS score ≥ 5, and periventricular lesion extension), with a score range of 0–3. In ROC curve analysis for the total study population, the risk score predicted a poor 6-month outcome with area under the curve (AUC) of 0.978 (95% CI: 0.950–1.000, *P* < .001) and 6-month mortality with AUC of 0.836 (95% CI: 0.745–0.927, *P* < .001, Fig. 2A and B). The risk score of 2 predicted a poor 6-month outcome with a sensitivity of 94.1% and a specificity of 95.2%, and 6-month mortality with a sensitivity of 93.3% and a specificity of 67.2%. For the subgroup without baseline encephalitis feature, the risk score predicted a poor 6-month outcome with AUC of 0.952 (95% CI: 0.896–1.000, *P* < .001) and 6-month mortality with AUC of 0.870 (95% CI: 0.764–0.978, *P* = .003, Fig. 2C and D). In this subgroup, the risk score of 2 predicted a poor 6-month outcome with a sensitivity of 85.7% and a specificity of 95.0%, and 6-month mortality with a sensitivity of 83.3% and a specificity of 81.2%.

**Figure 3:**
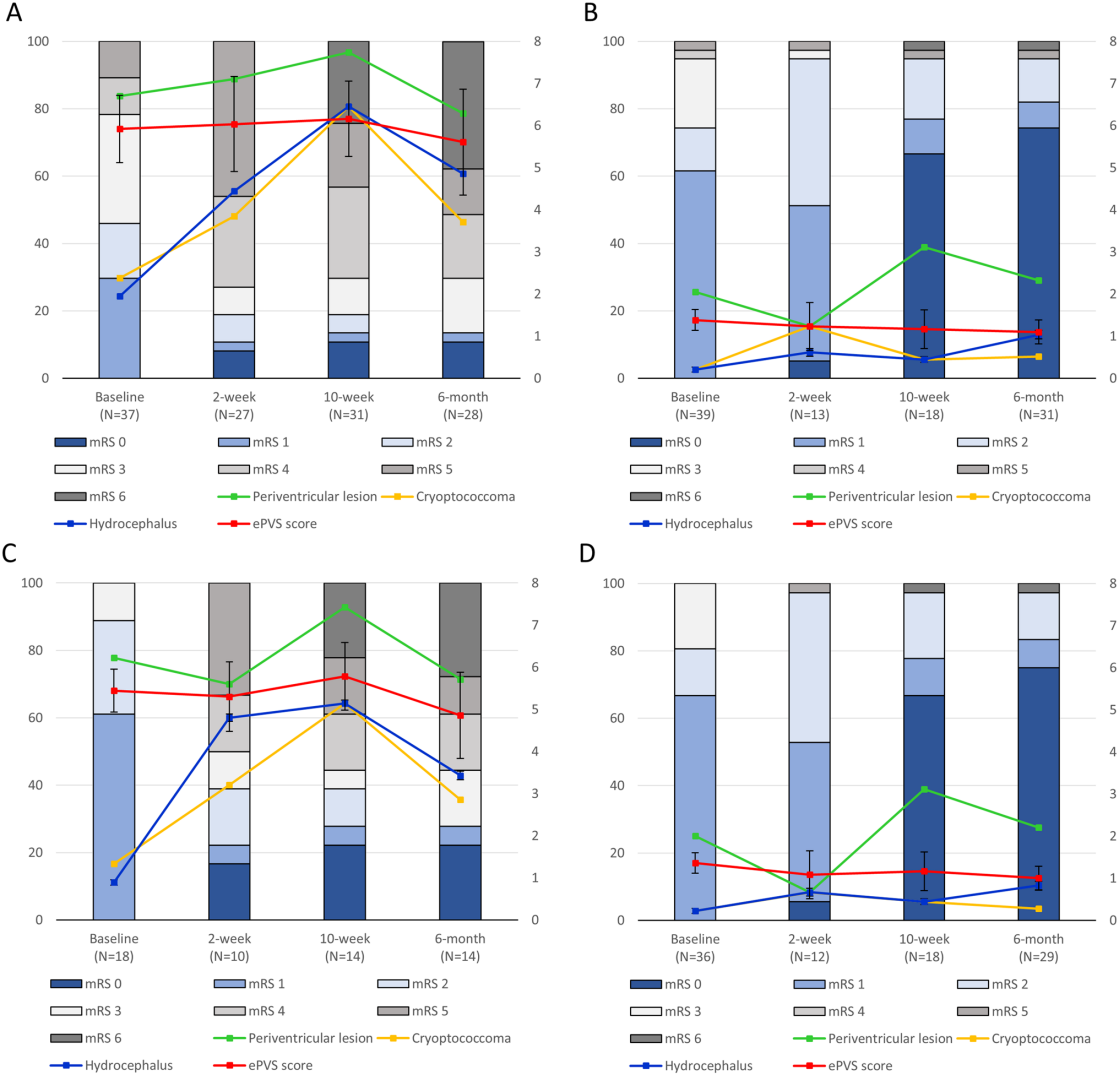
Serial changes in the MRI parameters and the modified Rankin scale (mRS) scores. Panel (**A**) demonstrates the subgroup with baseline ePVS score ≥ 5 (n = 37) and Panel (**B**) describes the subgroup with baseline ePVS score < 5 (n = 39) in the whole study population (n = 76). Panel (**C**) demonstrates the subgroup with baseline ePVS score ≥ 5 (n = 18) and Panel (**D**) describes the subgroup with baseline ePVS score < 5 (n = 36) in the subgroup without baseline encephalitis feature (n = 54). The bar graph indicates the modified Rankin scale (mRS) score profiles and the line graphs indicates the changes in the frequency of each MRI findings and the changes in the total enlarged perivascular space (ePVS)score. The numbers in the X axis indicates the number of patients included in the MRI analysis in each time point. Every patient was included in each time point of the mRS graph.

**Figure 4:**
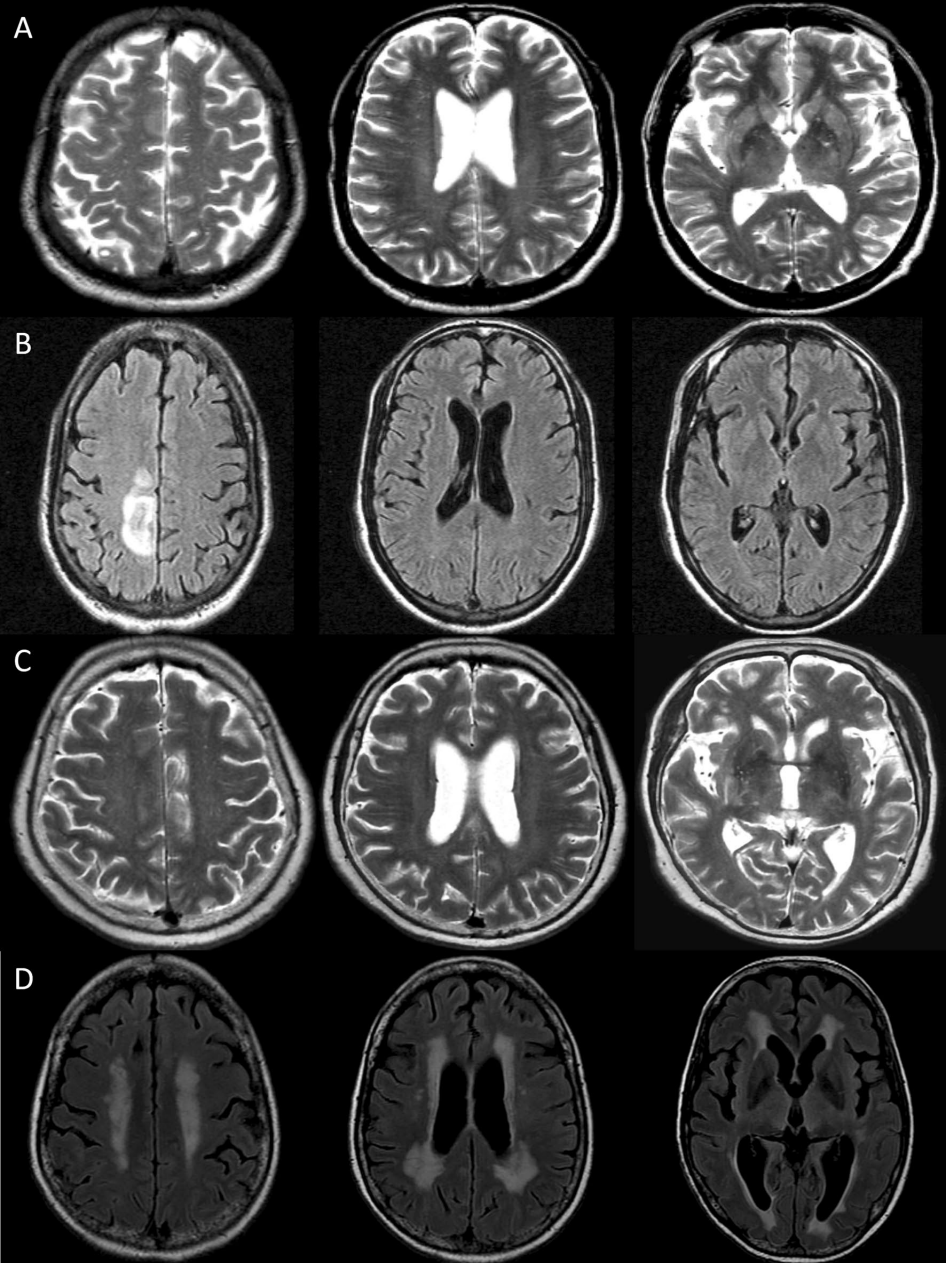
Illustrative cases. A 56-year-old man with underlying human immunodeficiency virus infection presented with fever and headache (initial modified Rankin Scale [mRS] score = 1). Baseline MRI showed enlarged periventricular space (EPVS) in centrum semiovale (CS-EPVS score = 4) and in basal ganglia (BG-EPVS score = 2, Panel (**A**)). The patient was treated with intravenous amphotericin combined with fluconazole for two weeks. However, follow-up MRI at 2 week showed interval development of parenchymal T2 high-signal intensity (HSI) at the right frontal cortex on T2 fluid attenuated inversion recovery (FLAIR) images (Panels (**B**)). Despite the amphotericin-based combination treatment for 40 days and oral fluconazole for 176 days, the mRS score at 6 month was 4. A 72-year-old immunocompetent woman presented with fever and headache (initial mRS score = 1). At baseline MRI, CS-EPVS score was 3 and BG-EPVS score was 2 (Panels (**C**)). The patient was treated with intravenous amphotericin combined with flucytosine and maintained with oral fluconazole for 214 days. However, gait disturbance and cognitive impairment progressed (6-month mRS score = 4). Follow-up MRI at 6 month showed interval development of periventricular extension of the T2 HSI and slight hydrocephalus on T2 FLAIR images (Evans ratio = 0.35, Panels (**D**)).

Two-week follow-up MRI evaluation data were available for 40 (59.7%), 10-week follow-up MRI for 49 (64.5%), and 6-month follow-up MRI for 59 (77.6%) patients. The median number of MRI evaluations analyzed per patient was 3 [3–3]. When the serial changes in the MRI parameters were analyzed in association with the changes in mRS scores, the subgroup with baseline ePVS score ≥ 5 showed gradual deterioration in the mRS score along with progressive increment of the frequency of cryptococcoma and hydrocephalus (Fig. 3A), whereas the subgroup with baseline ePVS score < 5 showed gradual improvement in the mRS score and maintained a low frequency of periventricular lesion extension, cryptococcoma, and hydrocephalus in the follow-up MRIs (Fig. 3B). A similar trend was observed in the subgroup without baseline encephalitis feature (Fig. 3C and D, see Fig. 4 for representative cases). The profiles of the MRI parameters between the groups evaluated using 1.5-T or 3.0-T MRI machines were comparable (Supplemental Table 4).

## Discussion

This study demonstrated clinical and MRI parameters associated with the progression and the poor outcomes of Cryptococcus meningoencephalitis. Along with the baseline encephalitis feature, a high ePVS score and periventricular lesion extension were independently associated with poor outcomes. Especially, presence of two or more risk factors at baseline showed high association with poor neurological outcomes and mortality, indicating that these might serve as prognostic markers. Given that the association was still valid for the subgroups without baseline encephalitis feature, these prognostic factors not only reflect the disease severity but also predict the risk of progression. Additionally, neurological deterioration manifested in brain MRI data as development of cryptococcoma and progression of hydrocephalus, which suggests that these MRI markers can also be used for monitoring the progression of disease.

According to a large-sized prospective study of 501 patients with HIV infection, high fungal burden in the CSF, altered mental status, old age, high peripheral WBC count, low body weight, anemia, and low CSF opening pressure were associated with 10-week mortality^1^. Further, combination treatment of amphotericin and flucytosine at induction period reduced 10-week mortality while fluconazole-based induction treatment was associated with higher mortality^3^. However, these studies mainly focused on mortality or fungal clearance in the CSF, while the neurological outcome of Cryptococcus meningoencephalitis has not been investigated in depth. In this regard, this is the first study to describe the dynamic neurologic course of the disease, and demonstrate the early accessible MRI factors that are useful to predict or monitor the neurological outcomes.

Notably, the outcome predictors in the current study reflect the distinct pathomechanism underlying the progression of Cryptococcus meningoencephalitis and can therefore be related with the previously reported prognostic factors for mortality. High fungal antigen titer and larger fungus capsule size might also contribute to a mechanical stagnation of CSF flow, especially in the peri-venular space which has small diameter^1,9,10^. Therefore, these factors can be correlated with the enlarged PVS in the baseline MRI which reflects the degree of CSF stasis. Altered mental status is a factor constituting a baseline encephalitis feature and is also related to MRI indicators of the parenchymal invasion of the Cryptococcus, such as periventricular lesion extension and cryptococcoma^1^. Increased ICP might also be the consequence of CSF recirculation failure resulting from the wide-spread cryptococcus accumulation over the perivascular space and manifest as the progression of hydrocephalus in MRI^8,9^.

In addition to outcome prediction and disease monitoring, the findings of the current study can also be useful for risk estimation and deciding the treatment strategy. For the patients with ≥ 2 baseline risk factors, higher combination or higher dose of anti-fungal treatment could be used to prevent the patient deterioration^25–27^. Additionally, frequent follow-up brain imaging to monitor the progression might be beneficial for the timely detection and early intervention to lower the ICP or other neurological complications.

The current study has several limitations. First, as a retrospective study, the number and the interval of CSF and MRI evaluations, and treatment regimen were heterogeneous and not standardized. Second, the study population with baseline MRI evaluation might bear a potential source of selection bias, as this criteria might exclude patients with severe or unstable baseline clinical status. Different subpopulations included in each time point of the serial MRI data analysis also warrant a careful interpretation of the result. Third, this study included both 1.5-T and 3.0-T machines, although baseline and follow-up images were obtained from the same scanner and the MRI parameter profiles between the groups with different MRI powers were comparable. Fourth, the rate of fungal clearance in CSF, one of the major parameters for determining treatment outcome, was not analyzed in this study^28^. Fifth, although the presence of pathological parenchyma contrast enhancement might be a potential outcome predictor as it represents a significant breakdown of blood–brain barrier, it was excluded from the outcome analysis due to the low frequency and the significant portion of patients without baseline T1 contrast enhancement images. Additionally, careful discrimination ePVS and periventricular lesion extension from aging-related cerebral small-vessel disease is warranted, although their associations with outcomes were significant after adjusting for age.

### Ethics approval

The design of this study was approved by the institutional review board of the Seoul National University Hospital.

## Supplementary Information

Supplementary Information.

## Data Availability

The datasets used and/or analysed during the current study are available from the corresponding author on reasonable request.
