# Author Correction: Enlarged periventricular space and periventricular lesion extension on baseline brain MRI predicts poor neurological outcomes in cryptococcus meningoencephalitis

**DOI:** 10.1038/s41598-021-97473-3

**Published:** 2021-08-31

**Authors:** Woo‑Jin Lee, Young Jin Ryu, Jangsup Moon, Soon‑Tae Lee, Keun‑Hwa Jung, Kyung‑Il Park, Manho Kim, Sang Kun Lee, Kon Chu

**Affiliations:** 1 Department of Neurology, Seoul National University Hospital, 101, Daehak‑ro, Jongno‑gu, Seoul, 110‑744 South Korea; 2 Laboratory for Neurotherapeutics, Center for Medical Innovations, Biomedical Research Institute, Seoul National University Hospital, Seoul, South Korea; 3 Department of Radiology, Seoul National University Bundang Hospital, Seongnam, South Korea; 4 Rare Disease Center, Seoul National University Hospital, Seoul, South Korea; 5 Department of Neurology, Seoul National University Hospital Healthcare System Gangnam Center, Seoul, South Korea; 6 Protein Metabolism Research Center, Seoul National University College of Medicine, Seoul, South Korea

Correction to: *Scientific Reports* 10.1038/s41598-021-85998-6, published online 19 March 2021

The original version of this Article contained an error, where informed consent information was incorrect.

As a result, in the Materials and Methods section, under the subheading ‘Study subjects’,

“Written informed consent was obtained from each patient or the patient’s legal surrogate.”

now reads:

“Informed consent was waived by the IRB, as this study did not intervene with the evaluation or treatment process of the patients, and the patients were anonymized during the study process.”

The original Article has been corrected.

