# Supplementary material for: Enlarged periventricular space and periventricular lesion extension on baseline brain MRI predicts poor neurological outcomes in cryptococcus meningoencephalitis
Source: Sci Rep. 2021 Mar 19;11:6446. doi: 10.1038/s41598-021-85998-6 (PMC7979784; doi:10.1038/s41598-021-85998-6)
Supplement: Supplementary file 1 — Supplementary Information. [file 41598_2021_85998_MOESM1_ESM.docx]

**Enlarged periventricular space and periventricular lesion extension on baseline brain MRI predicts poor neurological outcomes in Cryptococcus meningoencephalitis**

**Running head:** Cryptococcus meningoencephalitis outcomes

Woo-Jin Lee, MD,^1,2^ Young Jin Ryu, MD,^3^ Jangsup Moon, MD, PhD,^1,2,4^ Soon-Tae Lee, MD PhD,^1^ Keun-Hwa Jung, MD PhD,^1^ Kyung-Il Park, MD, PhD,^1,2,5^ Manho Kim, MD, PhD,^1,6^ Sang Kun Lee, MD, PhD,^1^ and Kon Chu MD, PhD^1,2^

^1^Department of Neurology, Seoul National University Hospital, Seoul, South Korea

^2^ Laboratory for Neurotherapeutics, Center for Medical Innovations, Biomedical Research Institute, Seoul National University Hospital, Seoul, South Korea

^3^ Department of Radiology, Seoul National University Bundang Hospital, Sungnam, South Korea

^4^ Rare Disease Center, Seoul National University Hospital, Seoul, South Korea

^5^ Department of Neurology, Seoul National University Hospital Healthcare System Gangnam Center, Seoul, South Korea

^6^ Protein Metabolism Research Center, Seoul National University College of Medicine, Seoul, South Korea

**Correspondence to:**

Kon Chu, MD, PhD, Department of Neurology, Seoul National University Hospital, 101, Daehak-ro, Jongno-gu, Seoul 110-744, South Korea. Tel: +82-2-2072-1878; Fax: +82-2-2072-7424; E-mail: stemcell.snu@gmail.com

**Counts:**

Number of characters

Title: 95, Running head: 42

Number of words

Abstract: 200, Manuscript body: 3352
Number of figures: 4, Number of tables: 3

Supplemental Materials

**List of Supplements: 4 Supplemental Tables**

**Supplemental Table 1.** MRI parameters for the MRI machines included in the analysis

**Supplemental Table 2.** Comparison of the clinical, laboratory, and treatment profiles between the groups with or without a baseline encephalitis feature.

**Supplemental Table 3.** Logistic regression models for poor 6-month outcomes in the subpopulation without baseline encephalitis feature

**Supplemental Table 4.** MRI parameters for the MRI machines included in the analysis

**Supplemental Table 1.** MRI parameters for the MRI machines included in the analysis

|  | GE Signa Excite HD 1.5T | GE Signa Excite 3.0T | GE Genesis Signa 1.5 | GE Discovery MR750w 3.0T | GE Signa Architect 3.0T | Philips Ingenia CX 3.0T | Siemens Skyra 3.0T | Siemens Magnetom vision plus 1.5T | Siemens Magnetom Sonata 1.5T | Siemens Magnetom Trio 3.0T |
| --- | --- | --- | --- | --- | --- | --- | --- | --- | --- | --- |
| FOV  (mm×mm) | 220×220 | 200×200 | 200×200 | 220×220 | 220×220 | 220×220 | 185×220 | 210×210 | 218×220 | 199×200 |
| FA | 90 | 90 | 90 | 142 | 150 | 90 | 150 | 180 | 150 | 140 |
| TR (ms) | 5000 | 9902 | 10002 | 8800 | 9000 | 9000 | 8000 | 9000 | 6000 | 9000 |
| TE (ms) | 108.2 | 168.5 | 123.5 | 89.2 | 92 | 110 | 105 | 119 | 112 | 97 |
| Thickness (mm) | 5 | 5 | 5 | 5 | 5 | 5 | 4 | 5 | 5 | 5 |
| Matrix | 448×256 | 320×192 | 512×140 | 288×288 | 256×256 | 352×225 | 384×204 | 512×126 | 448×400 | 384×209 |
| NEX | 2 | 2 | 1 | 2 | 1 | 1 | 1 | 1 | 2 | 1 |

GE: General electrics, HD: high definition, FOV: field-of-view, FA: flip angle, TR: repetition time, TE: echo time, and NEX: number of excitations.

**Supplemental Table 2.** Comparison of the clinical, laboratory, and treatment profiles between the groups with or without a baseline encephalitis feature.

| Parameters | **Encephalitis**  (n=22) | **Non-encephalitis**  (n=54) | ***P*** |
| --- | --- | --- | --- |
| **Demographic & Clinical profiles** |  |  |  |
| Female Sex (%) | 10 (45.5) | 18 (33.3) | 0.327 |
| Age of onset (years) | 56.1±16.3 | 55.1±14.5 | 0.801 |
| **Underlying Immune status** |  |  |  |
| Compromised | 11 (50.0) | 40 (74.1) | 0.111 |
| HIV infection | 2 (9.1) | 17 (31.5) | 0.015^*^ |
| Hematologic malignancy | 3 (13.6) | 9 (16.7) | 0.747 |
| Solid organ cancer | 2 (9.1) | 7 (13.0) | 0.641 |
| Post-transplant | 1 (4.5) | 3 (5.6) | 0.860 |
| High-dose immune suppressant | 3 (13.6) | 4 (7.4) | 0.401 |
| Immune competent | 11 (50.0) | 14 (25.9) | 0.111 |
| Fever >38.0°C | 20 (90.9) | 50 (92.6) | 0.808 |
| GCS<15 | 19 (86.4) | 21 (38.9) | <0.001^**^ |
| Seizure | 5 (22.7) | 2 (3.7) | 0.057 |
| mRS score | 3 [3–4.3] | 1 [1–2] | <0.001^**^ |
| **Laboratory profiles** |  |  |  |
| CSF protein level (mg/dL) | 108.5 [78.3–235.3] | 67 [50–83.2] | 0.067 |
| CSF WBC count (/uL) | 46.5 [19–90] | 46.5 [8–160] | 0.482 |
| CSF opening pressure (≥20 cmH2O) | 16 (72.7) | 16 (29.6) | <0.001^**^ |
| High CSF Ag titer (>1000) | 1 (4.5) | 1 (1.9) | 0.512 |
| **MRI profiles** |  |  |  |
| BG ePVS score | 3 [2–3] | 1 [0–2] | <0.001^**^ |
| CS ePVS score | 3.5 [3–4] | 1.5 [0–3] | <0.001^**^ |
| Total ePVS score | 6 [5–7] | 3 [0–5] | <0.001^**^ |
| Periventricular lesion extension | 18 (81.8) | 23 (42.6) | 0.001^**^ |
| Cryptococcoma | 8 (36.4) | 4 (7.4) | 0.015^*^ |
| Hydrocephalus | 7 (31.8) | 3 (5.6) | 0.021^*^ |
| **Treatment profiles** |  |  |  |
| Induction treatment |  |  |  |
| Amphotericin | 22 (100.0) | 54 (100.0) | 1.000 |
| Flucytosine | 12 (54.5) | 31 (57.4) | 0.822 |
| Fluconazole | 7 (31.8) | 16 (29.6) | 0.856 |
| Fluconazole consolidation + maintenance treatment | 22 (100.0) | 52 (96.3) | 0.383 |
| **Outcome profiles** |  |  |  |
| Antigen clearance at 2-weeks | 3 (13.6) | 25 (46.3) | 0.002^**^ |
| Antigen clearance at 10-weeks | 11 (50.0) | 39 (72.2) | 0.085 |
| mRS score at 10-weeks | 4 [3.8–5.3] | 0 [0–2] | <0.001^**^ |
| Mortality at 10-weeks | 5 (22.7) | 5 (9.3) | 0.187 |
| mRS score at 6-months | 4 [3.8–6] | 0 [0–3] | <0.001^**^ |
| mRS score >2 at 6-months | 20 (90.9) | 14 (25.9) | <0.001^**^ |
| Mortality at 6-months | 9 (40.9) | 6 (11.1) | 0.008^**^ |

Data are reported as mean ± standard deviation, or as median [interquartile range, IQR]. HIV: human immunodeficiency virus, GCS: Glasgow coma scale, mRS: modified Rankin scale, CSF: cerebrospinal fluid, WBC: white blood cell, and ePVS: enlarged perivascular space. ^*^ *P*<0.05 and ^**^ *P*<0.01.

**Supplemental Table 3.** Logistic regression models for poor 6-month outcomes in the subpopulation without baseline encephalitis feature

| Regression variables | Odd Ratio (95% CI) | *P* | R^2^ |
| --- | --- | --- | --- |
| **Model 1** |  |  | 0.745 |
| Age (years) | 0.946 (0.847−1.055) | 0.316 |  |
| ePVS score | 4.574 (1.431−14.62) | 0.010^*^ |  |
| Periventricular extension | 11.96 (0.868−164.718) | 0.064 |  |
| **Model 2** |  |  | 0.781 |
| Age (years) | 0.921 (0.803−1.055) | 0.235 |  |
| ePVS score ≥5 | 93.057 (4.805−1802.078) | 0.003^**^ |  |
| Periventricular extension | 49.679 (2.183−1130.353) | 0.014^*^ |  |

ePVS: enlarged perivascular space. ^*^ *P*<0.05 and ^**^ *P*<0.01.

**Supplemental Table 4.** MRI parameters for the MRI machines included in the analysis

|  | **3.0T**  (n=54) | **1.5T**  (n=22) | ***P*** |
| --- | --- | --- | --- |
| Female Sex (%) | 18 (33.3) | 10 (45.5) | 0.327 |
| Age of onset (years) | 55.3±15.5 | 55.7±13.9 | 0.906 |
| Baseline mRS score | 2 [1–3] | 1 [1–3] | 0.113 |
| **Baseline MRI profiles** | n=54 | n=22 |  |
| Total ePVS score | 4 [0.8–6] | 4.5 [1–6] | 0.701 |
| Periventricular lesion extension | 30 (55.6) | 11 (50.0) | 0.665 |
| Cryptococcoma | 9 (16.7) | 3 (13.6) | 0.747 |
| Hydrocephalus | 7 (13.0) | 3 (13.6) | 0.938 |
| **2-week MRI profiles** | n=30 | n=10 |  |
| Total ePVS score | 5 [0–7] | 5 [4.8–6] | 0.490 |
| Periventricular lesion extension | 20 (66.7) | 6 (60.0) | 0.711 |
| Cryptococcoma | 11 (36.7) | 4 (40.0) | 0.855 |
| Hydrocephalus | 11 (36.7) | 5 (50.0) | 0.469 |
| **10-week MRI profiles** | n=38 | n=11 |  |
| Total ePVS score | 6 [0–6] | 6 [4–7] | 0.100 |
| Periventricular lesion extension | 27 (71.1) | 10 (90.9) | 0.104 |
| Cryptococcoma | 19 (50.0) | 7 (63.6) | 0.435 |
| Hydrocephalus | 18 (47.4) | 8 (72.7) | 0.138 |
| **6-month MRI profiles** | n=44 | n=15 |  |
| Total ePVS score | 4 [0–5.8] | 4 [2–6] | 0.625 |
| Periventricular lesion extension | 24 (54.5) | 7 (46.7) | 0.605 |
| Cryptococcoma | 11 (25.0) | 4 (26.7) | 0.900 |
| Hydrocephalus | 16 (36.4) | 5 (33.3) | 0.836 |
| **Clinical outcomes** |  |  |  |
| mRS score at 6-months | 2 [0–4.3] | 1 [0–4.5] | 0.802 |
| Mortality at 6-months | 9 (16.7) | 6 (27.3) | 0.348 |

Data are reported as mean ± standard deviation, or as median [interquartile range, IQR]. mRS: modified Rankin scale and ePVS: enlarged perivascular space.
